# Research on imbalance machine learning methods for MR$T_1$WI soft tissue sarcoma data

**DOI:** 10.1186/s12880-022-00876-5

**Published:** 2022-08-26

**Authors:** Xuanxuan Liu, Li Guo, Hexiang Wang, Jia Guo, Shifeng Yang, Lisha Duan

**Affiliations:** 1 College of Computer Science and Technology, Qingdao University, Qingdao, 266071 China; 2 Department of Radiology, The Affiliated Hospital of Qingdao University, Qingdao, China; 3 Department of Radiology, Shandong Provincial Hospital Affiliated to Shandong First Medical University, Jinan, China; 4 Department of Radiology, The Third Hospital of Hebei Medical University, Shijiazhuang, Qingdao, China

**Keywords:** Soft tissue sarcoma, Radiomics, Machine learning, Extremely randomized trees, Imbalanced data

## Abstract

**Background:**

Soft tissue sarcoma is a rare and highly heterogeneous tumor in clinical practice. Pathological grading of the soft tissue sarcoma is a key factor in patient prognosis and treatment planning while the clinical data of soft tissue sarcoma are imbalanced. In this paper, we propose an effective solution to find the optimal imbalance machine learning model for predicting the classification of soft tissue sarcoma data.

**Methods:**

In this paper, a large number of features are first obtained based on $T_1$WI images using the radiomics methods.Then, we explore the methods of feature selection, sampling and classification, get 17 imbalance machine learning models based on the above features and performed extensive experiments to classify imbalanced soft tissue sarcoma data. Meanwhile, we used another dataset splitting method as well, which could improve the classification performance and verify the validity of the models.

**Results:**

The experimental results show that the combination of extremely randomized trees (ERT) classification algorithm using SMOTETomek and the recursive feature elimination technique (RFE) performs best compared to other methods. The accuracy of RFE+STT+ERT is 81.57% , which is close to the accuracy of biopsy, and the accuracy is 95.69% when using another dataset splitting method.

**Conclusion:**

Preoperative predicting pathological grade of soft tissue sarcoma in an accurate and noninvasive manner is essential. Our proposed machine learning method (RFE+STT+ERT) can make a positive contribution to solving the imbalanced data classification problem, which can favorably support the development of personalized treatment plans for soft tissue sarcoma patients.

## Background

Soft tissue sarcoma is a clinically rare and highly heterogeneous tumor, accounting for about 1% of all malignant tumors [1, 2]. Based on features such as histologic type and subtype, tumor necrosis and mitotic activity, French Federation Nationale des Centres de Lutte Contre le Cancer (FNCLCC) divides soft tissue sarcoma into grades I $\sim$ III [3]. In adults, histologic grading is the most important prognostic factor and the best indicator of the risk of metastasis in soft tissue sarcoma [3–5]. It is critical to patient prognosis and the development of treatment plans. Currently, biopsy is a primary method for obtaining pathologic grade preoperatively. But errors in biopsy may lead to inaccurate results due to tumor heterogeneity [3], especially in fatty tumors with large lesions [6]. Therefore, it is necessary to explore an accurate and non-invasive method for preoperative grading of soft tissue sarcoma.

In recent years, radiomics has been widely used for neoplastic lesions in various systems. Because of its objective and descriptive characteristics, it can analyse, refine and quantify medical images, so that the most valuable imaging features can be selected to analyze clinical information, differential diagnosis of tumors, and provide accurate guidance for treatment and prognosis [7, 8]. Previous studies have shown that MRI-based histological features are associated with pathological grade of soft tissue sarcoma [9].

For classification tasks on graded predictions of soft tissue sarcoma, the dataset is often imbalanced. That is, there is a class in the dataset that contains much more data than other classes. With the development of science and technology, in the current era of big data, more and more imbalanced data sets appear, so there is an urgent need for well-performing classifiers to accomplish such grading tasks. Ideally, the classifier can provide a better classification accuracy for both positive and negative examples. However, existing studies have shown that class imbalance will reduce the performance of some standard classifiers, such as decision trees, support vector machine, artificial neural networks, etc [10]. In fact, traditional classifiers usually have high classification accuracy for majority classes, while for minority classes, classification accuracy is very low. Taking the classification problem of soft tissue sarcoma as an illustration, if there are 1000 patients, 10 are positive examples (low grade), and 990 are negative (high grade). In this case, if the classifier maps all inputs as negative examples, the accuracy rate is as high as 99%. Obviously, this classifier is wrong and unusable, and the evaluation indicators are also not practicable. In recent years, researchers tend to pay more attention to the classification performance of the classifier for minority classes, such as medical diagnosis [11–14], bankruptcy prediction [15], natural disaster prediction [16], credit card fraud detection [17], anomaly detection [18], and so on. Using machine learning methods can overcome the problem of data imbalance, and achieve better results for medical data classification problems.

For the classification problem of imbalanced dataset, the solutions are divided into three categories [19–21]: (i) data level approaches, sampling the data to achieve the balance of the number of samples, undersampling and oversampling are generally the most common methods [19, 22]; (ii) algorithmic approaches, optimizing the algorithm to modify the conventional classification method to the situation of data imbalance, so that the improved conventional algorithm can have better results on imbalanced data [21]. (iii) cost-sensitive learning approaches, combining data level and algorithms to give higher costs on the minority classes in the sample that are classified incorrectly to achieve the final good results [19]. In this paper, we follow the first category approach to achieve excellent classify method on imbalance data, that is conventional methods are applied to classify the preprocessed data by oversampling and undersampling techniques.

When it comes to conventional classification method, researchers mainly use decision tree/random forest analyses and neural networks [23]. Some other popular machine learning methods are adapted to solve this kind problem, included support vector machine classifiers [24], latent growth mixture modeling [25], boosting methods [26] and so on.

In addition to the above methods, some specialized classification methods are designed for handling imbalanced data to achieve better result. Khalilia *et* *al*. combine repeated random subsampling with RF and predict disease risk from highly imbalanced data [27]. Majid *et* *al*. use K-nearest neighbors and support vector machines to predict human breast and colon cancer from imbalanced data [28] . Barot *et* *al*. propose an improved decision tree algorithm to diagnose Covid-19 [29] . Xie *et* *al*. propose a new data resampling technique called Gaussian Distribution based Oversampling (GDO), which combines SVM to classify imbalanced data [30]. Rustum *et* *al*. propose a hybrid resampling approach and combine the extra tree classifier to predict Pulsars [31]. Rupapara *et* *al*. propose an ensemble method called regression vector voting classifier (RVVC) for identifying the toxic comments on social media platforms [32]. Fatima *et* *al*. present three feature selection algorithms (RONS/ROS/ROA) to minimize the overlapping and perform fraud detection [33]. Rustum *et* *al*. adopt a deep neural network approach and propose a model named BIR (bleedy image recognizer) ,which combines the MobileNet with a custom-built convolutional neural network (CNN) model to classify the bleedy images of wireless capsule endoscopy [34]. Reshi *et* *al*. propose a deep CNN architecture for diagnosing COVID-19 based on the chest X-ray image classification [35]. Table 1 shows the specific methods. These are all effective ways to deal with imbalanced data, and achieve good results.

Notably, classification problems for medical imbalanced data usually do not work well with an individual machine learning method. In general, it is a common process to learning the data: performing feature selection, sampling it, and then classifying it with the specified classification method. This series of processes needs to be considered as a whole. Existing methods only consider classifiers or only improve classification methods, which are not effective in solving the soft tissue sarcoma grading problem. Such, we take researches accordingly in order to advance the implementation of imbalance learning.

**Table 1:** Summary of recent literature on solving data imbalance problems

Ref	Year	Dataset	Methods	Evaluation metric
[27]	2011	National Inpatient Sample (NIS) data	Repeated random subsampling-RF	AUC = 88.79%
[28]	2014	Real datasets of human protein	MTD-SVM	AC = 96.71%
[29]	2021	From Hospital Israelita Albert Einstein	MiDT	AC = 93.255%
[30]	2022	The esophageal cancer patient dataset	GDO-SVM	AUC = 0.71
[30]	2022	Wisconsin	GDO-SVM	AUC = 0.9662
[31]	2020	HTRU2	Hybrid resampling-ETC	AC = 99.3%
[32]	2021	The comments on social media platforms	RVVC-SMOTE	AC = 97%
[33]	2021	UCI(fraud detection)	RONS/ROS/ROA-LR/SVM	Gmean = 0.905
[34]	2021	WCE images	BIR-CNN	AC = 99.3%
[35]	2021	Chest X-ray image dataset	CNNs	AC = 99.5%

*AC* Accuracy; The datasets and evaluation measures in the table are selected from parts of the original literature or the best performing ones

In this paper, a feature dataset based on the MR$T_1$WI is first obtained by using radiomics methods, then different sampling and classification methods are adopted, and such that different machine learning models are composed for training the recursive feature elimination. We try to explore these machine learning methods and find an optimal one for predicting the pathological grading of soft tissue sarcoma. The main contributions of this paper are as follows:

1. This study explore multiple machine learning models with several well-known classification algorithms, such as extremely randomized trees (ERT), balanced random forest (BRF), random forest (RF), and support vector machine (SVM).
2. 252 MRI image data of soft tissue sarcoma are collected and processed in this study. A feature dataset is calculated after analyzing the images by recursive feature elimination (RFE). Resampling the imbalanced dataset with multiple sampling methods like random oversampling examples (ROSE), synthetic minority oversampling technique (SMOTE), SMOTETomek (STT) and adaptive synthetic sampling (ADASYN), are discussed here.
3. Different methods of feature selection, sampling and classification are combined, and extensive experiments are performed to classify imbalanced soft tissue sarcoma data. We find that the best one is RFE+STT+ERT. A dataset splitting method called SRS is used, which could improve the classification performance and verify the validity of the methods.

## Method

In this section, we first show the dataset used in the experiments then introduce the methods and reasons that we choose in feature selection method, sampling technology and classification algorithm in details. After that, we specifically explore effective classification methods for imbalanced soft tissue sarcoma data and present the training process of 17 different machine learning models. Furthermore, A dataset splitting method called SRS is used to verify the validity of the methods. The final, we show the evaluation metrics adopted for the experiments.

### **The dataset**

This paper uses preoperative MRI data of 252 patients with soft tissue sarcoma from January 2007 to the March 2018, 122 cases from the Affiliated Hospital of Qingdao University, 130 cases from Shandong Provincial Hospital Affiliated to Shandong First Medical University and The Third Hospital of Hebei Medical University. We name this dataset MRI-QSH. The dataset has following inclusion and exclusion criteria:

Inclusion criteria:

1. Histopathologically confirmed soft tissue sarcoma with complete clinical data after surgery;
2. Soft tissue sarcoma is graded according to the FNCLCC system (grade I $\sim$ III);
3. MRI scanning is performed within 2 weeks before treatment, and the cross-sectional $T_1$WI images were included.

Exclusion criteria:

1. Poor MRI image quality, signal-to-noise ratio $\le$1.0;
2. There are some other malignant tumors during treatment.

**Fig. 1:**
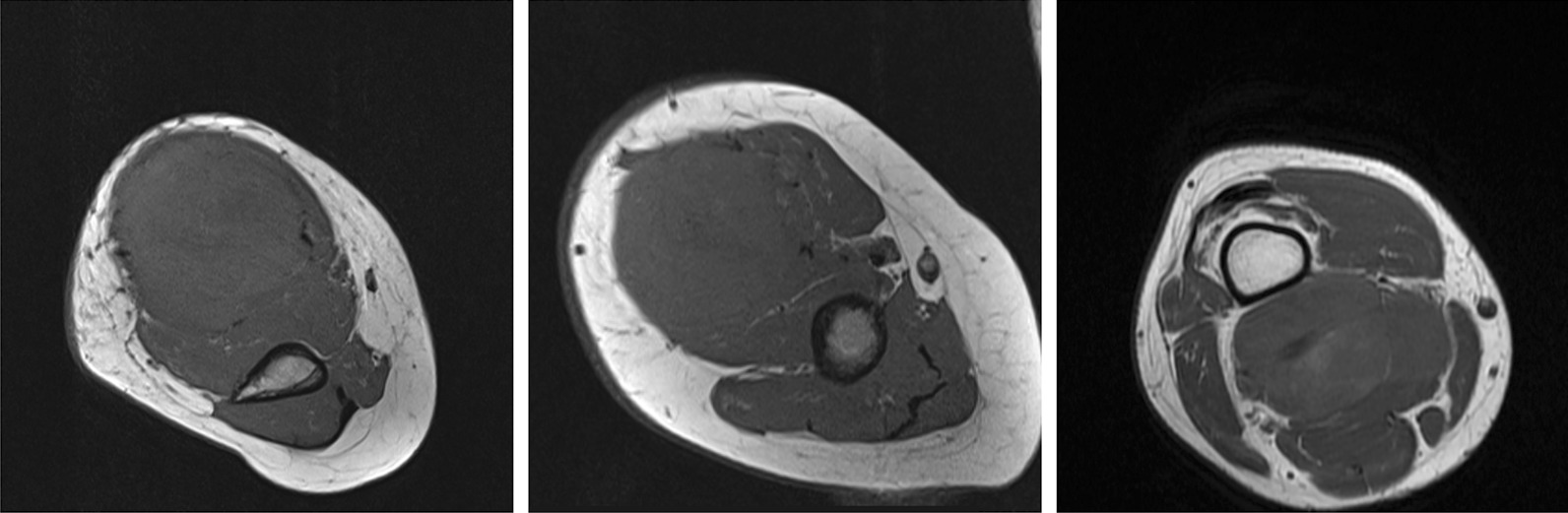
Example images of soft tissue sarcoma obtained by radiomics

According to the FNCLCC classification of soft tissue sarcoma data, grade I is low-grade, grade II and grade III are high-grade. The MRI-QSH dataset includes 62 patients with low-grade soft tissue sarcoma and 190 patients with high-grade soft tissue sarcoma. Table 2 shows the details of the number of high-grade and low-grade samples. Some selected soft tissue sarcoma images are shown in Fig. 1.

**Table 2:** The number of samples and the radio of the imbalance dataset in MRI-QSH dataset

The number of samples	Hige-grade	Low-grade	Imbalance ratio
252	190	62	3.06

By using MR scanning method, image segmentation, image standardization, and feature calculation, the features of MR$T_1$WI of MRI-QSH before machine learning methods were extracted by the company of Deepwise, such that we get the 2758 dimensional feature space dataset with 252 samples, and name it DW-QSH.

### **Feature selection method**

The data DW-QSH has a large number of feature parameters, 2758 in total, many of which do not contribute to the classification or have low contribution. Therefore, using feature selection will be beneficial to the accuracy of classification and can speed up the classification speed. For the feature selection method, this research chooses to use the recursive feature elimination (RFE) algorithm [36, 37]. RFE belongs to one of the packing method feature selection algorithms, which is a common method. RFE is used with a learner, which is usually a classifier. When we train the classifier, it sorts all the features and removes the ones that contribute the least to the classification. This process is performed recursively and is an example of reverse feature elimination. If removing some unnecessary features using RFE, it is more beneficial for the training of the model.

### **Data preprocessing**

In the dataset DW-QSH, there are 62 samples belonging low-grade and 190 samples belonging high-grade. It is an imbalance data learning problem that the number of high-grade samples is much larger than that of low-grade samples. In order to overcome the problem of data imbalance, we adapt some sampling methods for data preprocessing. There are three common sampling methods: (i) undersampling; (ii) oversampling; (iii) the combination of oversampling and undersampling. Due to the small amount of total data in this study, taking undersampling will cause the sample size to be further reduced and the sampling results will not be representative, which will also lead to less accurate final results. Therefore, we select some oversampling methods (random oversampling examples, synthetic minority oversampling technique and adaptive synthetic sampling) [38–40], and a combination method of oversampling and undersampling (SMOTETomek) [41].

#### Oversampling technique

Oversampling is to generate a minority of samples for imbalanced data to achieve data balance. Two oversampling methods are commonly used, that is the random oversampling examples method (ROSE) and the synthetic minority oversampling technique (SMOTE).

Random oversampling examples (ROSE) [39, 42, 43] randomly replicate samples from the minority class and add them to the training dataset, eventually making the number of minority classes equal to the number of majority classes, resulting in a new balanced dataset. Thus a single instance may be selected multiple times, and ROSE may increase the possibility of overfitting, but this sampling technique is very effective for machine learning algorithms that are subject to skewed distributions.

The synthetic minority oversampling technique (SMOTE) [38] is an improved method based on the random oversampling algorithm, where the minority class is oversampled by generating “synthetic” data rather than directly by replication. The basic idea of SMOTE is to analyze minority samples, artificially synthesize new samples based on minority samples, and add them to the dataset. However, influenced by the parameters, data distribution and other factors, the artificially generated data from the minority class may appear in the majority class, which will affect the final classification results.

Adaptive synthetic sampling (ADASYN) [40] is an improved method based on SOMTE. It assigns different weights to different minority classes of samples according to the data distribution, thus generating different numbers of new samples. ADASYN not only can reduce the learning bias caused by the imbalanced distribution of the original data, but also adaptively shifts the decision boundary to the difficult-to-learn samples. It has the disadvantage of being susceptible to outliers. If the K nearest neighbors of a minority class sample are all majority class samples, its weights become large and may generate noise.

#### The combination of oversampling and undersampling

In the SMOTE method, it is likely to generate some noise data when the boundary sample and the others are oversampled. It can be eliminated by cleaning the sample after oversampling. Tomek Link is an undersampling technique used to clean up overlapping samples. The synthetic minority oversampling technique+Tomek Link (SMOTETomek) [41] combines TomekLink and SMOTE, which is a combination of oversampling and undersampling.

### **Classification methods**

Random forest (RF) and support vector machine (SVM) are the most common classifiers in tumor image segmentation, tumor image classification and other applications [44]. In soft tissue sarcoma grading prediction problems, previous studies have shown that RF perform better than SVM [1]. In the following content, RF and its derivative methods are introduced in this subsection. For better comparison, SVM is also selected as one of the classification methods. We aim to explore the most effective classifier for the soft tissue sarcoma grade problem.

#### Random forest

Random forest [45] is a kind of ensemble learning, and its basic cell is decision tree. For each node of the decision tree, it has a put-back for sampling. For a sample set, it randomly selectes features to train and then uses the cart algorithm for calculation. This process is not pruned. For the classification of soft tissue sarcoma, each decision tree is a classifier and they perform classification independently. If there are *n* decision trees, then *n* classification results are generated. RF integrates all classification voting results and chooses the category with the most votes as the final output. RF is simple and easy to implement, suitable for handling imbalanced data, but not friendly for small data or low-dimensional datasets.

#### Balanced random forest

In the case of data imbalance, RF may contains a large number of majority classes and a small number of minority in the selected samples when building decision trees, and may favor the majority classes in the final classification vote. Balanced Random Forest (BRF) [46] combines the ideas of random undersampling and ensembing, where the majority of classes are undersampled and an equal number of minority classes are randomly selected for replacement, as a way to achieve a balanced training set. In the early stage of this study, sampling methods have been used to overcome the problem of data imbalance. Therefore, the performance of BRF in this experiment is not necessarily outperform random forest, but due to inconsistent sampling methods, the results achieved are also different. So we also select BRF as one of the classification methods to get the performance of each model combination.

#### Extremely randomized trees

Extremely randomized trees (ERT) [47] is an extension of RF. ERT is also an ensemble of decision trees, where each decision tree t $\in$ {1...T}, T is the number of decision trees. In the process of selecting data samples, ERT differs from RF in that each decision tree is independently trained using the entire data sample. In node partitioning, RF selects the optimal feature value to partition the points after searching in the feature subset, while ERT randomly selects features to partition the decision tree. ERT uses random features and random thresholds for partitioning.

For a given data point *x* and dataset $D_{train}$, a feature vector is represented by *f*(*x*,$D_{train}$). When classifying class *c* of the data, $p_t$ represents the conditional probability that the feature vector *f*(*x*,$D_{train}$) belongs to class *c*. For data point $x'$ , the probability that it belongs to class *c* is calculated by calculating the average of the probabilities on all trees [48] :

1 $$\begin{aligned} p(c|f(x,D_{train}))=\frac{1}{T}\sum _{t=1}^{T} p_t(c|f(x,D_{train})) \end{aligned}$$

Compared with RF, ERT makes the shape and difference of each decision tree larger and more random. In theory, the effect of generalization will also be better. The specific performance of the two classifiers will be obtained in the later experiments.

#### Support vector machine

Support Vector Machine (SVM) is a generalized linear classifier that performs binary classification of data by supervised learning. The basic model of SVM is to find the best separating hyperplane on the feature space that maximizes the positive and negative sample interval on the training set. SVM is applied in character recognition, facial recognition, pedestrian detection, text classification and other fields.

### **The state-of-the-art method**

One of the latest imbalanced data classification method called GDO-SVM [30] is used as the comparison. Xie *et* *al*. proposed an oversampling-based Gaussian distribution (GDO) that weights the minority class points by calculating their density information and distance information, probabilistically selecting anchor instances and generating new minority class instances based on the Gaussian distribution. After that, using SVM for Classification.

However, GDO-SVM is mainly an improvement on the sampling method, GDO-SVM performs well in KEEL and some public datasets of UCI, but from the performance of classifying real medical data listed in the literature, its improvement is not obviously good enough. The methods discussed in this paper are tackling this issue.

### **Model definition**

**Fig. 2:**
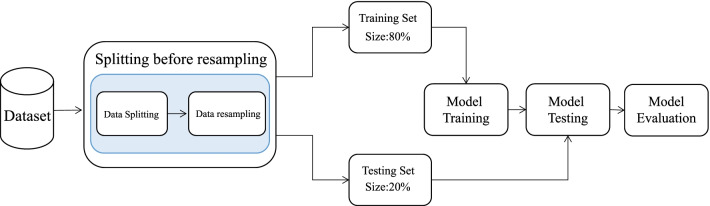
The conventional dataset splitting process

In the experiment, the feature selection method of RFE is applied. After that, selecting different sampling strategies and classification algorithms, and use the discard-one cross-validation method to obtain 16 different machine learning models and a state-of-the-art method, as shown in Table 3. The original data DW-QSH is divided into a “training set” and a “testing set” at a fixed ratio of 4 : 1. For each machine learning model, the “training set” and “testing set” are first divided on the dataset, perform resampling and model training on the “training set”, and verify the performance of the model on the “testing set”. Fig. 2 shows the specific process.

**Table 3:** 17 different machine learning models

Number	Feature selection method	Sampling technique	Classification method
1	RFE	ROSE	ERT
2	RFE	SMOTE	ERT
3	RFE	STT	ERT
4	RFE	ADASYN	ERT
5	RFE	ROSE	RF
6	RFE	SMOTE	RF
7	RFE	STT	RF
8	RFE	ADASYN	RF
9	RFE	ROSE	BRF
10	RFE	SMOTE	BRF
11	RFE	STT	BRF
12	RFE	ADASYN	BRF
13	RFE	ROSE	SVM
14	RFE	SMOTE	SVM
15	RFE	STT	SVM
16	RFE	ADASYN	SVM
17	RFE	GDO	SVM

*RFE* recursive feature elimination;* ROSE* random oversampling examples;* SMOTE* synthetic minority oversampling technique;* STT* SMOTETomek;* ADASYN* adaptive synthetic samping;* ERT* extremely randomized trees;* RF* random forest;* BRF* balanced random forest;* SVM* support vector machine

In order to ensure the validity of the results, for each model, we did 10 experiments with different “random state” in the process of splitting the dataset randomly, calculated the average and standard deviation of each evaluation metric.

### **A dataset spiltting method**

Due to the low-incidence of soft tissue sarcoma, it is very difficult to collect data, resulting in the number of samples is small. Meanwhile, the data is imbalanced, which is a greater challenge to train the model. It may cause that the classifier cannot identify the minority class samples (low-grade) well.

**Fig. 3:**
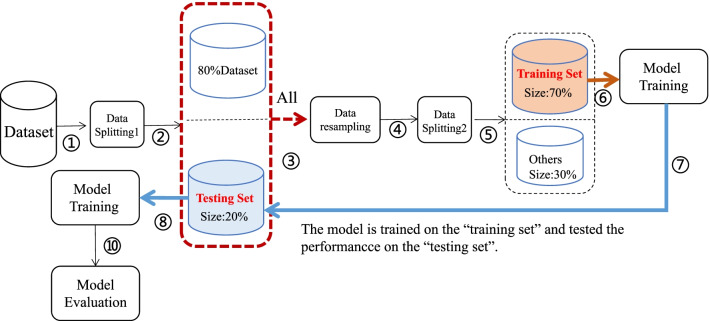
The process of dataset spitting with SRS

In order to better validate the performance of the models, we use the following dataset splitting method: firstly, 20% of the dataset is randomly divided into “testing set”, then the whole data set is oversampled, 70% of the oversampled data is randomly divided into “training set”. The classifier is trained on the ‘training set” and tested on the “testing set”, we call this method SRS. The detailed process is shown in Fig. 3.

### **Evaluation criteria and procedure**

To better evaluate the performance of the model, we use the following evaluation metrics: area under the curve (AUC) of the receiver operating characteristic (ROC), accuracy, specificity, sensitivity and G-mean of the model on predicting high-level and low-level soft tissue sarcomas in the experiment.

The ROC curve is a curve drawn with “True Positive Rate (TPR)” (reflecting the sensitivity of the classification result) as the ordinate, and “False Positive Rate (FPR)” (reflecting the specificity of the classification result) as the abscissa. “True Positive Rate” and “False Positive Rate” are derived from the “confusion matrix” of the classification results, as shown in Table 4. The rows are the predicted results, and the columns are the actual results. TP (True Positive) is the number of positive examples classified correctly, FN (False Negative) is the number of incorrectly classified negative examples, FP (False Positive) is the number of incorrectly classified positive examples, and TN (True Negative) is the correct number of negative examples.

Accuracy (*Acc*) is the ratio of the number of correctly classified instances to the total number of instances in the test set, which measures the classification ability of the model. G-mean is a composite metric for evaluating the accuracy of positive and negative instances for imbalanced data sets which consists of two subcomponents: Sensitivity (*Sens*) and Specificity (*Spec*). The following Eqs. (2)–(5) are given to describe these metrics.

**Table 4:** Confusion matrix of classification results

	Positive example	Negative example
Positive example	TP	FN
Negative example	FP	TN

2 $$\begin{aligned} Acc = \frac{TN+TP}{TP+FP+FN+TN} \times 100\% \end{aligned}$$

3 $$\begin{aligned} Sens = \frac{TP}{TP+FN}\times 100 \% \end{aligned}$$

4 $$\begin{aligned} Spec = \frac{TN}{FP+TN}\times 100 \% \end{aligned}$$

5 $$\begin{aligned} \mathrm{G-mean} =\sqrt{Sens \cdot Spec} \end{aligned}$$

Area Under Curve (AUC) is the area enclosed by the coordinate axis under the ROC curve and the area is always $\le$ 1. Meanwhile, since the ROC curve is generally located above the straight line *y*=*x*, the value of AUC ranges from 0.5 $\sim$ 1, which can be used as an indicator to evaluate the performance the model. The closer the AUC is to 1, the better the effectiveness of classifier is. When AUC=0.5, the model has no practical meaning.

## Results

### **Experimental results**

In the “testing set” of this study, various machine learning models exhibit different classification abilities. Since 10 different “random state” is selected and tested for each model when dividing the dataset, the performance of each model is eventually evaluated by taking the average of the metrics obtained from the 10 experiments.

#### Results on the conventional dataset spitting method

**Fig. 4:**
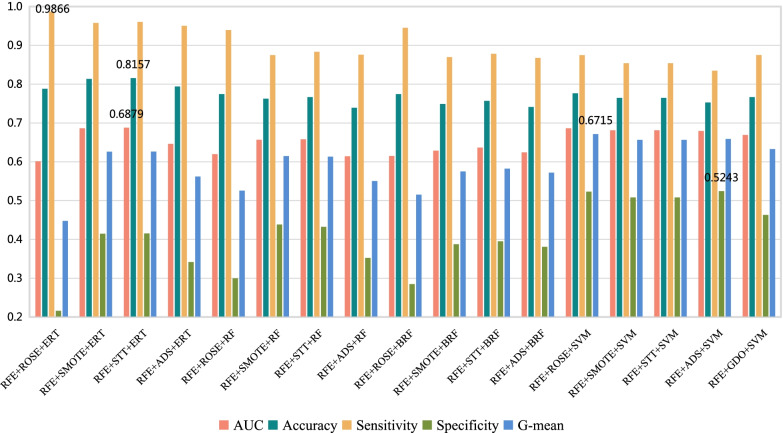
Histogram of classification performance of 17 models

Experiments are performed with the conventional dataset splitting method, and the results are shown in Table 5, $\sigma$ represents the standard deviation of 10 experiments. The histogram of results of 17 models is shown in Fig. 4, to compare the performance of each model on soft tissue sarcoma data classification prediction. It can be obtained that the ERT classification combined with RFE and STT technology (named Model 3) predicts the classification of soft tissue sarcoma data more effectively than others. The AUC, accuracy, sensitivity, specificity and G-mean of high-grade and low-grade soft tissue sarcomas of Model 3 are 0.6879, 81.57%, 96.03%, 41.55% and 0.6263, respectively. Even though the sensitivity (*Sens*) , specificity(*Spec*) and G-mean of Model 3 is not the best one among 17 models, but the AUC and Accuracy(*Acc*) perform best. Combining the performance of all evaluation metrics, Model 3 is the most effective model for identifying high and low grade of soft tissue sarcoma. The accuracy of RFE+STT+ERT is 81.57% , which is close to 82% by biopsy [49].

**Table 5:** The effectiveness of 17 different machine learning methods in the testing set

N	FS	ST	CM	AUC ± ${\sigma }$	*Acc*(%) ± ${\sigma }$	*Sens*(%) ± ${\sigma }$	*Spec*(%) ± ${\sigma }$	G-mean ± ${\sigma }$
1	RFE	ROSE	ERT	0.6013 ± 0.0482	78.82 ± 0.0545	**98.66** ± 0.0227	21.60 ± 0.1014	0.4477 ± 0.1154
2	RFE	SMOTE	ERT	0.6863 ± 0.0515	81.37 ± 0.0500	95.80 ± 0.0284	41.47 ± 0.0972	0.6260 ± 0.0782
3	RFE	STT	ERT	**0.6879** ± 0.0553	**81.57** ± 0.0533	96.03 ± 0.0254	41.55 ± 0.1091	0.6263 ± 0.0860
4	RFE	ADASYN	ERT	0.6461 ± 0.0595	79.41 ± 0.0464	95.04 ± 0.0279	34.18 ± 0.1121	0.5621 ± 0.1017
5	RFE	ROSE	RF	0.6197 ± 0.0473	77.45 ± 0.0533	93.97 ± 0.0425	29.97 ± 0.0865	0.5258 ± 0.0746
6	RFE	SMOTE	RF	0.6567 ± 0.0488	76.27 ± 0.0502	87.50 ± 0.0427	43.84 ± 0.1032	0.6147 ± 0.0700
7	RFE	STT	RF	0.6580 ± 0.0447	76.67 ± 0.0448	88.35 ± 0.0396	43.25 ± 0.1018	0.6133 ± 0.0680
8	RFE	ADASYN	RF	0.6142 ± 0.0618	73.92 ± 0.0599	87.60 ± 0.0482	35.24 ± 0.1026	0.5503 ± 0.0877
9	RFE	ROSE	BRF	0.6151 ± 0.0332	77.45 ± 0.0446	94.52 ± 0.0356	28.49 ± 0.0645	0.5154 ± 0.0593
10	RFE	SMOTE	BRF	0.6287 ± 0.0487	74.90 ± 0.0422	86.97 ± 0.0381	38.77 ± 0.1031	0.5750 ± 0.0770
11	RFE	STT	BRF	0.6367 ± 0.0578	75.69 ± 0.0436	87.84 ± 0.0461	39.51 ± 0.1182	0.5822 ± 0.0872
12	RFE	ADASYN	BRF	0.6243 ± 0.0331	74.12 ± 0.0441	86.76 ± 0.0370	38.10 ± 0.0735	0.5720 ± 0.0503
13	RFE	ROSE	SVM	0.6863 ± 0.2226	77.65 ± 0.0436	87.49 ± 0.0438	52.30 ± 0.1295	**0.6715** ± 0.0789
14	RFE	SMOTE	SVM	0.6812 ± 0.0591	76.47 ± 0.0606	85.41 ± 0.0633	50.82 ± 0.0894	0.6564 ± 0.0672
15	RFE	STT	SVM	0.6812 ± 0.0591	76.47 ± 0.0606	85.41 ± 0.0633	50.82 ± 0.0894	0.6564 ± 0.0672
16	RFE	ADASYN	SVM	0.6795 ± 0.0483	75.29 ± 0.0499	83.48 ± 0.0672	**52.43** ± 0.0815	0.6588 ± 0.0557
17	RFE	GDO	SVM	0.6691 ± 0.0685	76.67 ± 0.0657	87.51 ± 0.0557	46.30 ± 0.1083	0.6328 ± 0.0580

Best results are highlighted in bold style

*N* number; *FS* feature selection; *ST* sampling technique; *CM* classification method; *AUC* area under the curve; *Sens* sensitivity; *Spec* specificity; *ROSE* random oversampling examples; *SMOTE* synthetic minority oversampling technique; *STT* SMOTETomek; *ADASYN* adaptive synthetic sampling; *RFE* recursive feature elimination; *ERT* extremely randomized trees; *RF* random forest; *BRF* balanced random forest; *SVM* support vector machine

#### Results on the SRS dataset spitting method

**Fig. 5:**
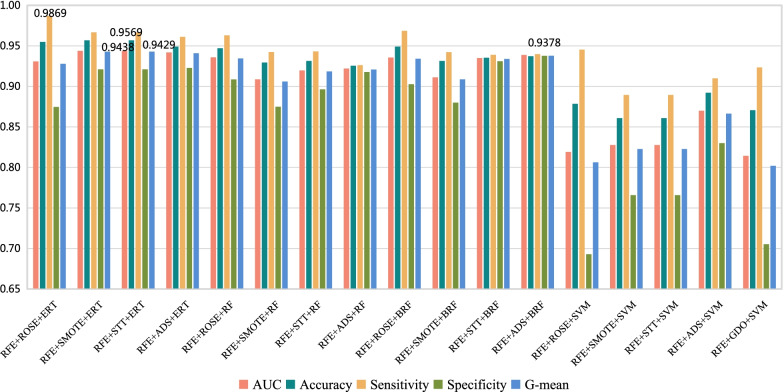
Histogram of classification performance of 17 models using the SRS method

Experiments are performed again, using the SRS dataset splitting memthod, and the performance of the 17 models is shown in Table 6. The histogram of classification performance of 17 models using the SRS method is shown in Fig. 5. Obviously, after using SRS method, the performance of all models has been improved, especially the specificity (*Spec*). Models 2 and 3 performed best, with the same results in ten experiments. The AUC, accuracy, sensitivity, specificity and G-mean of high-grade and low-grade soft tissue sarcomas of Model 3 are 0.9438, 95.69%, 96.66%, 92.10% and 0.9429, respectively. Even the sensitivity (*Sens*) and specificity (*Spec*) are not the best, but the sensitivity (*Sens*) is close to 98.69% and the specificity (*Spec*) is clost to 93.78%.

**Table 6:** Performance of the SRS dataset splitting method on 17 models in the testing set

N	FS	ST	CM	AUC ± ${\sigma }$	*Acc*(%) ± ${\sigma }$	*Sens*(%) ± ${\sigma }$	*Spec*(%) ± ${\sigma }$	G-mean ± ${\sigma }$
1	RFE	ROSE	ERT	0.9308 ± 0.0445	95.49 ± 0.0245	**98.69** ± 0.0139	87.47 ± 0.0915	0.9278 ± 0.0478
2	RFE	SMOTE	ERT	**0.9438** ± 0.0382	**95.69** ± 0.0203	96.66 ± 0.0229	92.10 ± 0.0713	**0.9429** ± 0.0391
3	RFE	STT	ERT	**0.9438** ± 0.0382	**95.69** ± 0.0203	96.66 ± 0.0229	92.10 ± 0.0713	**0.9429** ± 0.0391
4	RFE	ADASYN	ERT	0.9419 ± 0.0430	94.90 ± 0.0280	96.11 ± 0.0200	92.28 ± 0.0821	0.9409 ± 0.0443
5	RFE	ROSE	RF	0.9358 ± 0.0410	94.71 ± 0.0321	96.30 ± 0.0363	90.86 ± 0.0754	0.9345 ± 0.0425
6	RFE	SMOTE	RF	0.9087 ± 0.0547	92.94 ± 0.0211	94.24 ± 0.0326	87.49 ± 0.1104	0.9059 ± 0.0600
7	RFE	STT	RF	0.9197 ± 0.0439	93.14 ± 0.0349	94.31 ± 0.0420	89.63 ± 0.0759	0.9185 ± 0.0447
8	RFE	ADASYN	RF	0.9220 ± 0.0429	92.55 ± 0.0289	92.62 ± 0.0322	91.78 ± 0.0865	0.9208 ± 0.0437
9	RFE	ROSE	BRF	0.9356 ± 0.0396	94.90 ± 0.0295	96.86 ± 0.0324	90.27 ± 0.0749	0.9342 ± 0.0412
10	RFE	SMOTE	BRF	0.9111 ± 0.0562	93.14 ± 0.0212	94.23 ± 0.0229	88.00 ± 0.1095	0.9088 ± 0.0614
11	RFE	STT	BRF	0.9350 ± 0.0284	93.53 ± 0.0186	93.89 ± 0.0315	93.10 ± 0.0695	0.9339 ± 0.0291
12	RFE	ADASYN	BRF	0.9388 ± 0.0404	93.73 ± 0.0304	93.99 ± 0.0346	**93.78** ± 0.0791	0.9378 ± 0.0415
13	RFE	ROSE	SVM	0.8191 ± 0.0448	87.84 ± 0.0180	94.53 ± 0.0324	69.29 ± 0.1085	0.8062 ± 0.0559
14	RFE	SMOTE	SVM	0.8276 ± 0.0545	86.08 ± 0.0339	88.94 ± 0.0445	76.59 ± 0.1120	0.8227 ± 0.0586
15	RFE	STT	SVM	0.8276 ± 0.0545	86.08 ± 0.0339	88.94 ± 0.0445	76.59 ± 0.1120	0.8227 ± 0.0586
16	RFE	ADASYN	SVM	0.8699 ± 0.0573	89.22 ± 0.0349	90.99 ± 0.0374	83.00 ± 0.1204	0.8664 ± 0.0604
17	RFE	GDO	SVM	0.8143 ± 0.0598	87.06 ± 0.0230	92.34 ± 0.0373	70.52 ± 0.1395	0.8020 ± 0.0752

Best results are highlighted in bold style

*N* number; *FS* feature selection; *ST* sampling technique; *CM* classification method; *AUC* area under the curve; *Sens* sensitivity; Spec: specificity; *ROSE* random oversampling examples; *SMOTE* synthetic minority oversampling technique; *STT* SMOTETomek; *ADASYN* adaptive synthetic RFEsampling; *RFE* recursive feature elimination; *ERT*extremely randomized trees; *RF*random forest; *BRF* balanced random forest; *SVM* support vector machine

In the experiment, SRS dataset splitting method can verify the effectiveness of the models. In general, Model 3 (RFE+STT+ERT) is the most effective method for predicting the grade of soft tissue sarcoma and it is better than the GDO-SVM. Since the whole process for classifying soft tissue sarcoma data is designed as: performing feature selection, sampling it, and then classifying it with the specified classification method, GDO-SVM only improves the sampling method, and for the data in this study, the performance of this method is not as good as RFE+STT+ERT.

### **Running time**

**Fig. 6:**
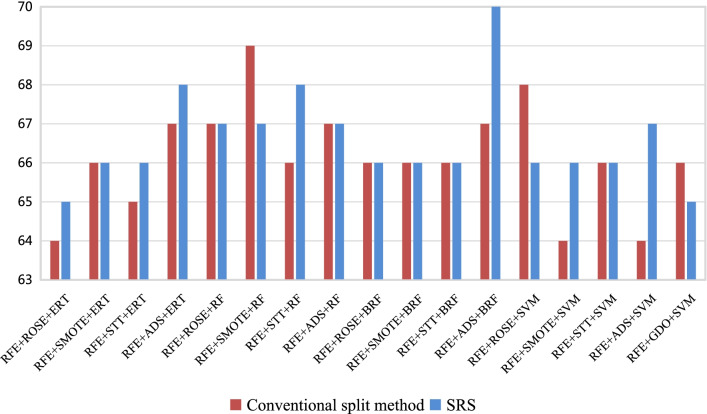
Running time of different machine learning models using different dataset spiltting methods

In addition to those above evaluation metrics, the running time is employed to compare the performance of different models on the DW-QSH. In measuring the running time, each model is subjected to 10 experiments individually (each “random state” is a test) , and the value is taken average value to obtain the final running time in seconds. Table 7 and Fig. 6 show the final results, the running times of the two different dataset splits are shown.

**Table 7:** Running time of different machine learning models

Number	Model	Conventional Split-Running time (s)	SRS-Running time (s)
1	RFE+ROSE+ERT	64	65
2	RFE+SMOTE+ERT	66	66
3	RFE+STT+ERT	65	66
4	RFE+ADASYN+ERT	67	68
5	RFE+ROSE+RF	67	67
6	RFE+SMOTE+RF	69	67
7	RFE+STT+RF	66	68
8	RFE+ADASYN+RF	67	67
9	RFE+ROSE+BRF	66	66
10	RFE+SMOTE+BRF	66	66
11	RFE+STT+BRF	66	66
12	RFE+ADASYN+BRF	67	70
13	RFE+ROSE+SVM	68	66
14	RFE+SMOTE+SVM	64	66
15	RFE+STT+SVM	66	66
16	RFE+ADASYN+SVM	64	67
17	RFE+GDO+SVM	66	65

*RFE* recursive feature elimination; * ROSE* random oversampling examples; * SMOTE* synthetic minority oversampling technique; * STT* SMOTETomek; * ADASYN* adaptive synthetic samping; * ERT* extremely randomized trees; * RF* random forest; * BRF* balanced random forest; * SVM* support vector machine

It can be obtained that the running time of each model has little difference, the method of dataset splitting has little effect on the running time of the model. The running time is around 66s, the highest is 70s, and the lowest is 64s. Since the running times of the 17 models differs little, the method with the best performance can be chosen, which is RFE+STT+ERT.

## Discussion

### **Impact of this article**

According to the study in this paper, the imbalance machine learning model of the combination of extremely randomized trees classification algorithm using SMOTETomk and recursive feature elimination technique, that is RFE+STT+ERT, performs best in classification prediction on the MR$T_1$WI soft tissue sarcoma data. In the future, we will further explore the performance of classifying other imbalanced data by this model, discuss more effective model on solving different medical data imbalance problems.

### Model performance when using SRS for dataset splitting

In the conventional dataset splitting method, the amount of low-grade data is small, so the models cannot resample valuable samples well during training, resulting in the classifier cannot identify low-grade soft tissue sarcoma well.

After using SRS, the performance of all models is improved. Because resampling is performed on the entire dataset, the classifier learns more characteristics of low-grade soft tissue sarcoma during training. Therefore, the performance of the models improves quickly, especially the specificity. Meanwhile, only 70% of the resampled data are selected to split in the training, and the testing set keep independent to prevent a large amount of data in the training set and the testing set being repeated. The validity of the experiment is guaranteed.

### **Study limitations**

There are some shortcomings in this paper as well. During the experiment, the data adopted is relatively small because soft tissue sarcomas are rare and not easy to obtain in practise. If a larger amount of valid data can be collected, it will better validate the classification efficiency of the machine learning model proposed in this paper. The obtained feature dataset DW-QSH is high-dimension, because we do not use a specified and targeted feature extraction method. Such that, we will explore to find an optimal feature extraction method for the present data to enhance the performance of imbalance machine learning model in the future.

## Conclusions

This paper analysis some imbalance machine learning approaches on classifying soft tissue sarcoma data, and aims to find a best research method for the pathological garding problem of soft tissue sarcoma. Firstly, based on the MR$T_1$WI radiomics, a large number of features are obtained as a feature dataset DW-QSH. Then, we explore the combinations of different sampling techniques, feature selection methods, and classification algorithms, and get nine imbalance machine learning models based on the DW-QSH. We also used a dataset splitting method called SRS, which can verify the effectiveness of the models. The experimental results show that the combination of RFE+STT+ERT performs best compared to other combination methods, even better than the state-of-the-art GDO-SVM method. The receiver operating characteristic area under the curve, accuracy, sensitivity, specificity and G-mean of this method for predicting high-grade versus low-grade soft tissue sarcoma are 0.6879, 81.57%, 96.03%, 41.55% and 0.6263. The accuracy of RFE+STT+ERT is 81.57% , which is close to 82% by biopsy. Meanwhile the value is 0.9438, 95.69%, 96.66% 92.10% and 0.9429 by using SRS, respectively. The running time of the method is about 66 seconds.

The classification results of this method are similar to those of the pre-surgical biopsy puncture, which means that the explored machine learning method has high research value for the classification of soft tissue sarcomas data. Therefore, it can provide useful support for developing personalized treatment plans for soft tissue sarcoma patients before surgery.

## Data Availability

The data used during the current study are available from the Hexiang Wang on reasonable request, email: wanghexiang@qdu.edu.cn. The URL of the code: https://github.com/Ally509/Code-Lxx-ML_FOR_MRT1WI

## Abbreviations

N: Number
FS: Feature selection
ST: Sampling technique
CM: Classification method
AUC: Area under the curve
Sens: Sensitivity
Spec: Specificity
ROSE: Random oversampling examples
SMOTE: Synthetic minority oversampling technique
STT: SMOTETomek
ADS: Adaptive synthetic sampling
ADASYN: Adaptive synthetic sampling
RFE: Recursive feature elimination
ERT: Extremely randomized trees
RF: Random forest
BRF: Balanced random forest
SVM: Support vector machine
